# The conditional approach to evaluating detection performance

**DOI:** 10.3758/s13414-021-02362-6

**Published:** 2021-10-08

**Authors:** Wolf Schwarz

**Affiliations:** Department of Psychology, University of Potsdam, P.O. Box 60 15 53, D – 14415, Potsdam, Germany

**Keywords:** Signal detection, Conditional inference, Odds ratio, Noncentral hypergeometric distribution, Fisher’s exact test, Likelihood-based confidence intervals

## Abstract

In many applied single-point Yes/No signal-detection studies, the main interest is to evaluate the observer’s sensitivity, based on the observed rates of hits and false alarms. For example, Kostopoulou, Nurek, Cantarella et al. (2019, *Medical Decision Making, 39,* 21–31) presented general practitioners (GPs) with clinical vignettes of patients showing various cancer-related symptoms, and asked them to decide if urgent referral was required; the standard discrimination index *d′* was calculated for each GP. An alternative conditional approach to statistical inference emphasizes explicitly the conditional nature of the inferences drawn, and argues on the basis of the response marginal (the number of “yes” responses) that was actually observed. It is closely related to, for example, Fisher’s exact test or the Rasch model in item response theory which have long been valuable and prominent in psychology. The conditional framework applied to single-point Yes/No detection studies is based on the noncentral hypergeometric sampling distribution and permits, for samples of any size, exact inference because it eliminates nuisance (i.e., bias) parameters by conditioning. We describe in detail how the conditional approach leads to conditional maximum likelihood sample estimates of sensitivity, and to exact confidence intervals for the underlying (log) odds ratio. We relate the conditional approach to classical (logistic) detection models also leading to analyses of the odds ratio, compare its statistical power to that of the unconditional approach, and conclude by discussing some of its pros and cons.

## Introduction

Signal detection theory (SDT) is one of the most successful methodological developments originating (Tanner & Swets, 1954) from psychology; it has pro foundly influenced theorizing and data analysis in many fundamental and applied fields in the behavioral sciences (for detailed background and review, see Green & Swets, 1966; Macmillan & Creelman, 2005; McNicol, 2005; Wickens, 2002; Wixted, 2020; for some more critical views, see Green, 2020; Mueller & Weidemann, 2008; Trimmer et al., 2017).

One of SDT’s most prominent applications is based on the data format shown in Table 1, often called the Yes/No (YN) design (Macmillan & Creelman, 2005, Ch. 1–2).

**Table 1:** Notation used to summarize the single-point detection design

stimulus	“yes”	“no”	sum
*s*_1_ *s*_0_	*x* *m* − *x*	*n*_1_ – *x* *n*_0_ – *m* + *x*	*n*_1_ *n*_0_
sum	*m*	*n*_0_ + *n*_1_ − *m*	*n*_0_ + *n*_1_

The signal stimulus *s*_1_ is presented in *n*_1_ trials, the noise stimulus *s*_0_ in *n*_0_ trials. In all *n*_0_ + *n*_1_ trials the observer has given a total of *m* “yes” responses, and thus *n*_0_ + *n*_1_ − *m* “no” responses. Of all *m* “yes” responses, *x* were given in signal trials, *m* − *x* in noise trials. The observed hit rate is *H* = *x*/*n*_1_, the observed false alarm rate is *F* = (*m* − *x*)/*n*_0_. The column totals *m* and *n*_0_ + *n*_1_ − *m* are called the response marginal, the row totals *n*_1_ and *n*_0_ form the stimulus marginal

In the YN design generating the data format shown in Table 1, an observer is presented in each trial with either a signal (*s*_1_) or a noise (*s*_0_) stimulus, and indicates his/her decision about the nature of the stimulus presented by responding “yes” or “no”; the Table represents a standard summary of potential results. Especially in applied settings the trial numbers *n*_0_, *n*_1_ per observer and condition of interest are often quite small (typical sample sizes in applied studies are *n*_0_ = *n*_1_ = 10 as, e.g., in Köteles et al., 2013, or *n*_0_ = *n*_1_ = 20 as, e.g., in O’Connor et al., 2003), and the main interest often centers on whether the observer’s ability to detect or discriminate the signals under study is better than chance and if so, by how much. Varying the bias of the observer towards one decision or the other provides additional information, but in many applied studies extracting detection indices, only a single pair of observed hit (*H*) and false alarm (*F*) rates is obtained, and the present note focuses on this widely used “single-point design” (e.g., Rotello et al., 2008).

A traditional and prominent approach for analyzing data in the format of Table 1 is to extract estimates of the sensitivity and bias measures *d′* and *c* (for detailed expositions, see, e.g., Macmillan & Creelman, 2005, Ch, 1–2; Stanislaw & Todorov, 1999) which are derived from the classical SDT model assuming internal stimulus representations which are normally distributed with equal variance^1^. In this model, *d′* is the separation of the means of the distributions, and c is the location of the decision criterion, relative to these means. Formally, this analysis may be interpreted as a transformation of the two independent probabilities *π*_*H*_ = P("*yes*"|s_1_) and *π*_*F*_ = P("*yes*"|s_0_) into the two indices *d′* and *c*, which carry information about conceptually separate aspects characterizing the performance of the observer, at least within the framework of the classical model. Even though *c* and *d* describe conceptually separate performance aspects their sample estimates are not independent^2^, whereas those of π_*H*_ and π_*F*_ (that is, *H* and *F*) are.

The present article focuses on the “small sample” research tradition (for detailed review, see Miller & Schwarz, 2018) in which sensitivity and bias measures are typically estimated for each observer separately, and then aggregated informally. This tradition is especially prominent, for example, in psychophysical and perceptual studies using practiced observers, in behavioristic research using few well-trained animals, or in neuropsychological studies of specific syndromes. In other research areas a *d′* or *c* score is computed separately for each participant in each of two conditions, and the mean scores are compared using, for example, a dependent *t* test. In this “large sample” research tradition, information about the standard errors, for example, of each observer’s *d′* estimate can improve statistical power by partitioning the total error variance used by *t* tests into a generic (systematic) between-subject component vs. a component due to pure sampling error of the estimates (Miller & Schwarz, 2018, Eq. 1).

The notion that an observer is unable to discriminate signal and noise corresponds to the assertion that the observed rates *H* and *F* differ only due to sampling error, that is, that the underlying true probabilities π_*H*_ and π_*F*_ are identical. In the context of the double-binomial YN sampling scheme generating Table 1, this assertion corresponds to the basic hypothesis about the equality of two independent probabilities, the statistical evaluation of which has generated a large literature. As described below, two^3^ prominent broad frameworks can be distinguished in the statistical literature: conditional and unconditional approaches. Given the correspondence between the notion of no discriminability and the hypothesis π_*H*_ = π_*F*_ mentioned above it is surprising that in evaluating detection performance exclusively one of these approaches (viz., the unconditional) has been used so far, especially since in many other areas of psychology examples of the conditional inference framework such as Fisher’s exact test (e.g., Hays, 1963, Ch. 17; McNemar, 1962, Ch. 13) or the Rasch (1966) model in item response theory have been prominent for a long time. The central aim of the present note is to present and to illustrate the alternative conditional approach to evaluating detection performance in the YN design. More specifically, we describe the conceptual framework on which the conditional approach rests, and illustrate some aspects of its technical application in the context of exemplary data in the format of Table 1; we relate this approach to classical detection models (Luce, 1959) also leading to analyses of the log odds ratio, compare its statistical power with that of the unconditional approach, and conclude by discussing some pros and cons of the conditional approach so as to indicate specific contexts where this approach is especially valuable.

### The conditional approach

Table 2 illustrates two potential outcomes of a detection task involving *n*_1_ = 14 signal and *n*_0_ = 14 noise trials. In the first scenario (Table 2, left) the hit and false alarm rates are *H* = 10/14, and *F* = 4/14 respectively, leading to the estimates $\hat{d^{\prime }}=1.13\  and\ \hat{c}=0$. The traditional analysis (Gourevitch & Galanter, 1967, Eq. 6; Macmillan & Creelman, 2005, Eq. 13.4) produces a standard error *SE* (*d′*) = 0.50; assuming a roughly normal sampling distribution of $\hat{d^{\prime }}$, the approximate 95% confidence interval for *d′* is equal to $\hat{d^{\prime }}$ ± 1.96 *SE* ($\hat{d^{\prime }}$) = [+0.15, 2.20]. For example, based on this confidence interval we would typically conclude that the detection performance in Table 2 (left) is better than chance; the width of the interval is 2.05.

**Table 2:**
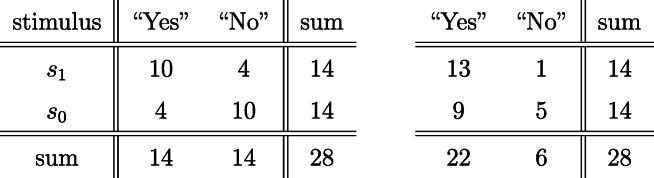
Two potential outcomes of a detection task involving 14 signal and 14 noise trials. In the first scenario (left Table) the hit and false alarm rates are *H* = 10/14, and *F* = 4/14, respectively, in the second scenario (right Table) these rates are *H* = 13/14, and *F* = 9/14. Both scenarios lead essentially to the same estimate of *d′*

Confidence intervals like [+0.15, 2.20] for Table 2 (left) are based on a first-order linearization (the so-called Delta method; e.g., Agresti, 2013, Ch. 16; Fleiss et al., 2003, Ch. 2.6; Pawitan, 2013, Ch. 4.7; Schwarz, 2008, pp. 110ff), and on assuming a normal sampling distribution of the estimate, $\hat{d^{\prime }}$. Both assumptions are reasonable for large samples but for small and medium samples these approximations can be poor, especially when the probabilities involved are close to zero or one. Based on the independent-binomial sampling scheme that underlies Table 1, these inaccuracies have been documented by detailed numerical and simulation results (see Hautus, 1995; Kadlec, 1999;  Macmillan, Rotello, & Miller, 2004; Miller, 1996; Verde et al., 2006).

A critical aspect of the traditional analysis is that statements about *d′* are conditional (i.e., dependent) on the specific value of the response bias ($\hat{c}$) that was actually observed. However, even though in the traditional approach the sampling distribution of $\hat{d^{\prime }}$ depends on the value of $\hat{c}$ this dependence is not reflected in the analysis carried out. That is, the traditional analysis does not formally condition on the observed response marginal, and thus misleadingly suggests that its conclusions (e.g., the standard error of *d′* ) are independent of the response marginal actually observed. It should thus be emphasized that the qualification “conditional” refers to the critical fact that the analysis in the approach so labeled is explicitly based on conditioning on the response marginal that was actually observed. To illustrate these points, we ask, more specifically: In exactly which sense are statements about *d′* conditional on the value of $\hat{c}$ observed?

Suppose the same observer (i.e., unchanged sensitivity) had used a laxer response criterion instead, leading to a higher hit rate *H* = 13/14, but also to more false alarms, *F* = 9/14 (Table 2, right). These values give essentially the same estimate of *d′* (i.e., $\hat{d^{\prime }}$ = 1.10) but a clearly laxer estimated response criterion of $\hat{c}$ = −0.92. The same traditional analysis used before now yields the considerably larger standard error of $\hat{d^{\prime }}$ equal to 0.61, an increase of 22% relative to an unbiased response criterion, so that the 95% confidence interval for *d′* widens to [−0.10, 2.30]. For example, based on this confidence interval we would typically conclude that the detection performance in Table 2 (right) is not significantly better than chance. In this sense, our conclusion regarding the sensitivity of an observer depends on which response criterion s/he happened to choose.

This example demonstrates the more general fact that the observed response marginal determines the precision with which conclusions about *d′* can be drawn. Specifically, the standard error of $\hat{d^{\prime }}$ and the width of the confidence interval depend on the observed number *m* of positive responses, and thus on the specific value of the “nuisance” parameter, *c*. The main argument advanced by the conditional framework is that it is thus appropriate to argue conditionally on the total number *m* of “yes” responses (or, in the context of the equal-variance normal model, on the value of $\hat{c}$) actually observed. This is to ensure that we attach to our conclusions regarding the comparison of hit and false alarm rates the precision actually achieved, and not that to be achieved hypothetically in distinct scenarios (i.e., with different response criteria) that have in fact not occurred. The conditional framework emphasizes explicitly the conditional nature of the inferences drawn. In addition, this approach permits explicit exact inference even in the case of arbitrarily small numbers of signal and noise trials.

**Fig. 1:**
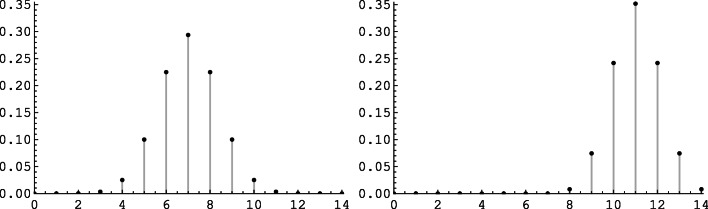
The central hypergeometric distribution. Left panel: out of a total of *n*_1_ = 14 signal and *n*_0_ = 14 noise presentations *m* = 14 trials are selected at random for a “yes” response (cf. Table 2, left). The abscissa shows the number of hits, the ordinate the associated hypergeometric probability. With probability 94.3% would the number of hits fall into the interval [5, 9]. Right panel: same scenario, but for *m* = 22 trials with a “yes” response (cf. Table 2, right), in which case the number of hits can only range from 8 to 14

To motivate the conditional approach consider a scenario involving an observer with no sensitivity (i.e., π_*H*_ = π_*F*_ ) for the signal in question; evaluating the hypothesis of no sensitivity lies at the heart of many detection studies. Suppose that before entering the lab this observer was determined to respond equally often “yes” and “no” (e.g., Kantner & Lindsay, 2012). In the scenario considered in Table 2, this completely insensitive observer would then distribute at random 14 “yes” responses among the total of 28 trials, much as drawing at random and without replacement 14 balls from an urn containing 14 red (signal) and 14 black (noise) balls. Any hypothetical replication of the design in Table 2 involving this unbiased observer would again produce 14 “yes” responses. In this scenario, the probability of scoring *x* hits (and thus 14 − *x* false alarms) is given by the central hypergeometric distribution shown in Fig. 1 (left). For example, assuming the observer is completely insensitive, the probability to get more than 9 or less than 5 hits (cf. Table 2, left) is equal to 0.057.

The above argument is an example of a more general statistical framework known as conditional inference (e.g., Agresti, 2013, Ch. 3.5 and 16.5; Cox & Snell, 1989, Ch. 2; Fleiss et al., 2003, Ch. 6; Pawitan, 2013, Ch. 10). As a simple scenario (Cox, 1958) illustrating its logic consider a two-stage chance experiment, in which, first, a fair coin is flipped. With “heads,” four values from a (*μ*, 1) normal distribution are drawn, and with “tails,” we draw 10,000 values from the same distribution; our aim is to estimate *μ* and to state a standard error for this estimate. Clearly, the observed sample mean $\overline{x}$ is in any case our best estimate of *μ*. Assume we are told that the coin has fallen tails—what, then, is the standard error of our estimate?

The standard unconditional approach ignores the specific outcome of the coin toss, and considers $\overline{x}$ to be one realization of a mixture of two equiprobable normal distributions of sample means, both having mean *μ*, but one with a large variance of 1/4, and one with a small variance of 1/10,000. After all, across many hypothetical independent replications of this experiment, the sample mean would be highly variable, reflecting mainly the large variance of $\overline{x}$ from those realizations in which the coin fell heads, when the sample size was only four. In this view, the variance (the squared standard error) of the estimate $\overline{x}$ across many hypothetical replications is close to 1/8; in particular, it is much larger than 1/10,000.

In contrast, the conditional inference approach takes the specific realization of the coin toss into account, and so the squared standard error would be equal to 1/10,000. After all, if we already know that the observed mean was based, specifically, on a sample of 10,000 values, then why should we ignore this critical information in forming the standard error, and weigh in hypothetical cases (i.e., had the coin fallen heads, the sample size would have been only 4) of which we already know that they had not occurred?

The situation with respect to the precision with which we can estimate sensitivity in a detection task is in several ways analogous to this more extreme example. Considering the two scenarios in Table 2, the coin toss corresponds to the choice of a neutral (left) vs. lax (right) response criterion, which in turn determines the response marginals in Table 2. The outcome of the coin toss (i.e., the choice of the response criterion) does not bias^4^ our estimate of *d′* , just as in the coin-toss example $\overline{x}$ remains the best estimate of μ for both heads or tails. However, as seen above from the associated confidence intervals, the response marginal has considerable influence on the precision of the estimate $\hat{d^{\prime }}$, just as the outcome of the coin toss influences the precision of the estimate $\overline{x}$. Within the conditional inference framework, this outcome is explicitly taken into account by conditioning on the response marginal that was actually observed.

### The conditional approach: Statistical framework

We denote as $\uplambda =\frac{\pi_H}{1-{\pi}_H}/\frac{\pi_F}{1-{\pi}_F}$ the odds ratio of the underlying hit and false alarm probabilities. For the single–point YN–design a standard result (Agresti, 2013, Eq. 7.9; Cox & Snell, 1989, Eq. 2.46; Fleiss et al., 2003, Eq. 6.35; Pawitan, 2013, Eq. 10.2) is that conditional on the observed response marginal the number x of hits has the non-central hypergeometric distribution

1 $$ \mathrm{P}\left(x;\uplambda, {n}_0,{n}_1\left|m\right.\right)=\frac{\left(\begin{array}{c}{n}_1\\ {}x\end{array}\right)\left(\begin{array}{c}{n}_0\\ {}m-x\end{array}\right){\uplambda}^x}{\sum_{i=\max \left(0,m\hbox{-} {n}_0\right)}^{\min \left({n}_1,m\right)}\left(\begin{array}{c}{n}_1\\ {}i\end{array}\right)\left(\begin{array}{c}{n}_0\\ {}m-i\end{array}\right){\uplambda}^i} $$

where *n*_1_ and *n*_0_ are the number of signal and noise trials, *m* is the total number of “yes” responses, and *x* is the number of hits. Note that in the layout of Table 1 the number of hits *x* must be at least 0 or *m* − *n*_0_, whichever is larger, and is at most *n*_1_ or *m*, whichever is smaller. The most remarkable feature of Eq. 1 is that it depends only on the odds ratio *λ* of the two independent probabilities π_*H*_ , π_*F*_ . For an observer with no sensitivity we have π_*H*_ = π_*F*_ , in which case the odds ratio is *λ* = 1, and Eq. 1 represents the central hypergeometric distribution.

In the following we denote as *ψ* = ln *λ* the log odds ratio. The log likelihood of the data *x* in Table 1, conditional on the response marginal *m*, is given as

2 $$ {\mathrm{L}}_m\left(\psi |x\right)=\ln\ \mathrm{P}\left(x;{e}^{\psi },{n}_0,{n}_1|m\right). $$

The conditional maximum likelihood estimate of *ψ* is the value that, for given *m* and observed *x*, maximizes *L*_*m*_(*ψ*| *x*) (e.g., Agresti, 2013, Ch. 16.4.4). Note that *L*_*m*_(*ψ*| *x*) is well-defined for any potential outcome *x*; in particular, it is well defined for *x* = *n*_1_ (when the observed hit rate is *H* = 1) and for *x* = *m* (when the observed false alarm rate is *F* = 0).

**Fig. 2:**
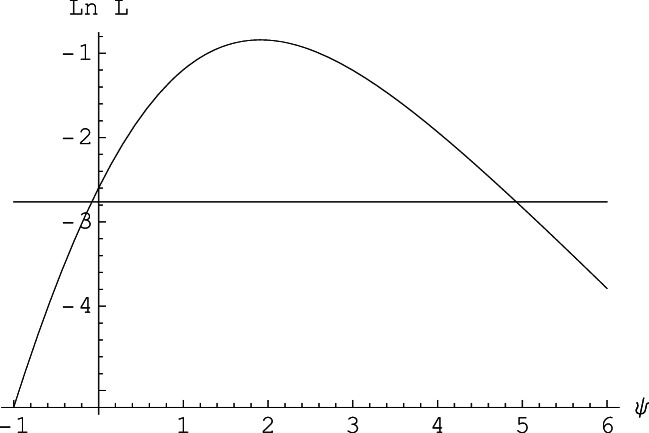
The log likelihood function *L*_*m*_(*ψ*| *x*) for *n*_0_ = *n*_1_ = 14, *m* = 22 and *x* = 13, giving *H* = 13/14 and *F* = 9/14 (cf. Table 2, right). The maximum occurs at $\hat{\psi}=1.91$, corresponding to an odds ratio of $\hat{\uplambda}=6.75$. The horizontal line lies $\frac{1}{2}{\chi}_{(1)}^2$ (0.95) = 1.92 units below the maximum of *L*_*m*_(*ψ*| *x*)

Figure 2 shows *L*_*m*_(*ψ*| *x*) for the data in Table 2 (right), when the observer gave in *n*_1_ = 14 signal and *n*_0_ = 14 noise trials a total of *m* = 22 “yes” responses, of which *x* = 13 were hits, and thus 9 false alarms. The value of $\hat{\psi}=1.91$ maximizes the conditional likelihood, and it corresponds to an odds ratio of $\hat{\uplambda}=6.75$.

Is the estimated value $\hat{\psi}=1.91$ compatible with the notion that the observer has no sensitivity to detect the signal (π_*H*_ = π_*F*_ )? Note that an outcome as in Table 2 (right) is difficult to evaluate with the standard technique because the assumptions underlying its application are clearly violated (i.e., *n*_1_, *n*_0_ are both small, and *H* is close to 1). In contrast, the conditional approach offers exact explicit solutions based on Eq. 1. Specifically, exact confidence intervals for *ψ* can be found analogously to the classical Clopper–Pearson intervals for binomial parameters (e.g., Agresti, 2013, Ch. 16.6; Miller, 1996, Eq. 12). The lower limit of these confidence intervals is obtained by finding the value of *ψ* for which a number of hits at least as large as the one observed (i.e., *x*) still has a probability of *α*/2. Similarly, an upper limit is obtained by finding the value of *ψ* for which a number of hits equal to or smaller than the one observed still has a probability of *α*/2.

More formally, define

3 $$ {b}_l\left(\psi \right)=\sum \limits_{i=x}^{\min \left({n}_1,m\right)}\mathrm{P}\left(i;{e}^{\psi },{n}_0,\left.{n}_1\right|m\right) $$

4 $$ {b}_u\left(\psi \right)=\sum \limits_{i=\max \left(0,m-{n}_0\right)}^x\mathrm{P}\left(i;{e}^{\psi },{n}_0,\left.{n}_1\right|m\right) $$

**Fig. 3:**
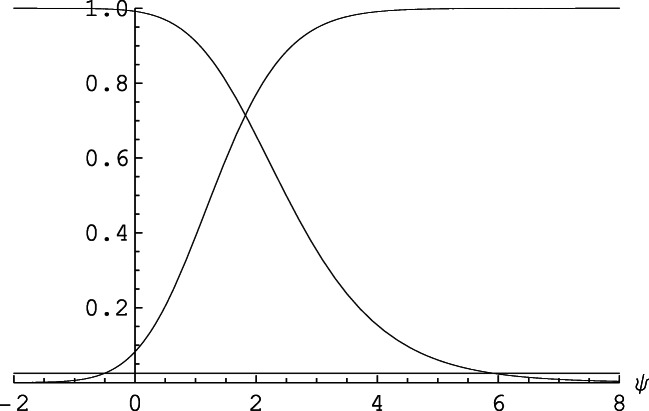
The functions *b*_*l*_ (*ψ*) (increasing) and *b*_*u*_(*ψ*) (decreasing) used to construct a confidence interval for the log odds ratio. For the data given in Table 2 (right), and *α* = 0.05, the limits are [−0.50, 5.91]. The confidence interval comprises all values of *ψ* for which both *b*_*l*_ (*ψ*) and *b*_*u*_(*ψ*) lie above α/2, a level indicated by the horizontal line at the bottom

For the data in Table 2 (right) the functions *b*_*l*_ (*ψ*), *b*_*u*_(*ψ*) are shown in Fig. 3; by its definition, *b*_*l*_ (*ψ*) must be increasing, and *b*_*u*_(*ψ*) be decreasing in *ψ*. Then the lower and upper boundary of the (central) (1 − *α*) confidence interval [*ψ*_*l*_, *ψ*_*u*_] are defined by the solutions of

5 $$ {b}_l\left(\left(\psi \right)\right)=\upalpha /2\ \mathrm{and}\ {b}_u\left(\left(\psi \right)\right)=\upalpha /2 $$

Intervals constructed in the manner of Eq. 5 shown in Fig. 3 guarantee a coverage probability for the log odds ratio (or equivalently for the odds ratio) of at least 1 − *α*. For the data in Table 2 (right), we obtain a 95% confidence interval for *ψ* equal to [−0.50, 5.91], which corresponds to a 95% confidence interval for λ of [0.61, 368.71]. The fact that this interval for *ψ* includes the value of zero indicates that the hypothesis of no sensitivity, π_*H*_ = π_F_ , cannot be ruled out (and thus possibly λ = 1). The width of the interval reflects the considerable uncertainty of inferences about λ when trial numbers as small as *n*_*1*_ = *n*_*0*_ = 14 are used.

Rather than inverting two one-sided tests as by Eq. 5, Agresti and Min (2001; Baptista & Pike, 1977) have shown that shorter confidence intervals are obtained by inverting one two-sided test (also see Agresti, 2013, Ch. 16). In effect, with this method the confidence interval consists of all values *ψ* for which the conditional probability *P*(*x*; *e*^*ψ*^, *n*_0_, *n*_1_| *m*) in Eq. 1 of the observed number *x* of hits, plus that of all values *x*′ with smaller probability together is smaller than α. For example, for the data in Table 2 (right) we obtain the shorter 95% confidence interval [−0.45, 5.20] for *ψ*, corresponding to [0.64, 181.27] for *λ*.

Finally, a graphical way to construct an approximate confidence interval is based on the likelihood ratio test of the observed vs. alternative values of *ψ*, see Morgan (2009, Ch. 4.4) or Pawitan (2013, Ch. 9). By this diagnostic, values of *ψ* for which the log likelihood function *L*_*m*_(*ψ*| *x*) shown in Fig. 2 falls more than $\frac{1}{2}{\chi}_{(1)}^2\left(1-\upalpha \right)$ units below the maximum *L*_*m*_(*ψ*| *x*) would be considered incompatible with the observed data at a level of α. The horizontal line in Fig. 2 indicates that level for α = .05 (i.e., $\frac{1}{2}{\chi}_{(1)}^2$ (0.95) = 1.92), leading to an approximate 95% confidence interval for *ψ* of [−0.08, 4.93]. This interval is only approximate because the likelihood ratio test is only asymptotically exact; the advantage of the diagnostic relative to the two exact methods described above is that it allows a quick and easy evaluation directly from the graph of the log likelihood function.

Finally, we emphasize that for all three methods described confidence intervals for *ψ* translate one-to-one into confidence intervals for the odds ratio λ by transforming the lower and upper interval boundaries via *λ* = exp(*ψ*). For example, intervals containing the value *ψ* = 0 translate into intervals containing the odds ratio λ = 1, corresponding to π_*H*_ = π_*F*_.

### The relation of the conditional approach to Luce’s choice model

**Fig. 4:**
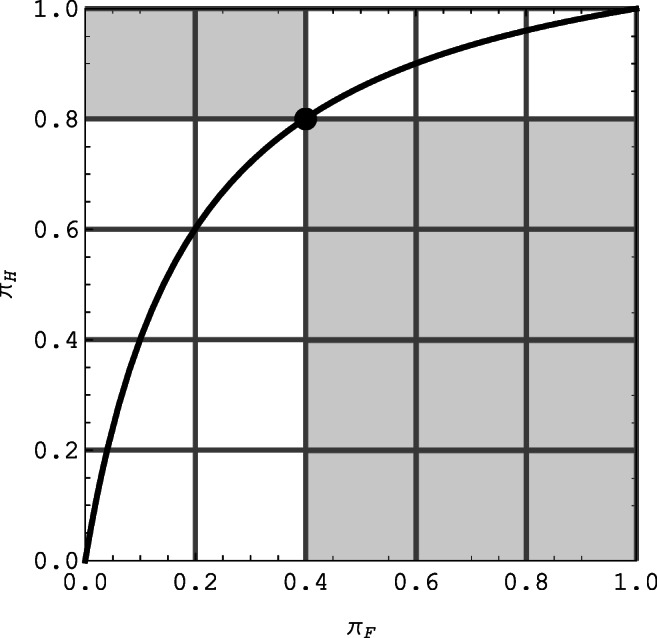
The locus of pairs (π_*F*_, π_*H*_ ) leading to a constant odds ratio, shown for *λ* = 6, corresponding to *ψ* = 1.79. For any point on the ROC curve λ equals the shaded area (12 subsquares) in the lower-right corner measured as a multiple of the shaded area (2 subsquares) in the upper-left corner

As indicated by Eq. 1 the conditional approach naturally leads to the odds ratio $\uplambda =\frac{\pi_H}{1-{\pi}_H}/\frac{\pi_F}{1-{\pi}_F}$ as a measure of sensitivity, or to functions of λ, such as *ψ*. One specific interpretation that also leads to the log odds ratio as a sensitivity index is the detection model based on Luce’s (1959; Macmillan & Creelman, 2005, ch. 4; McNicol, 2005, ch. 6) logistic choice model. In this model the internal stimulus representation X_n_ under noise has a logistic distribution with mean −*d*′/2, whereas the stimulus representation X_s_ for signals has a logistic distribution with mean +*d*′/2. The observer gives a positive response if X_*n*_ or if X_*s*_ exceeds the response criterion c, leading to a false alarm or a hit, respectively. Under these assumptions, the log odds ratio *ψ* is equal to *d′*, that is, to the standard logistic discrimination index, measuring the separation of the two logistic densities (cf., McNicol, 2005, Eq. 6.9). In the framework of Luce’s logistic model, varying the response criterion c for a given, fixed separation *d′* = ln λ traces out an isosensitivity curve; it is given by the parametric family $x\mapsto y(x)=\frac{\uplambda_{\times }}{\left(1-\times \right)+{\uplambda}_{\times }}$, and shown in Fig. 4. The ROC curve *y(x)* has the property that the associated odds ratio $\frac{y}{1-y}/\frac{x}{1-x}$ for all of its points *(x, y)* remains constant at λ. Figure 4 illustrates a geometric interpretation of the odds ratio: for any point on the ROC curve λ equals the shaded area in the lower–right corner measured as a multiple of the shaded area in the upper-left corner.

Whereas the logistic choice model can be thought of as one specific processing mechanism generating data conforming to a given odds ratio, we note that the use of the odds ratio as described in the previous Section encompasses a wider range of conceptual models capable of generating the observed results. Specifically, under the design shown in Table 1, the distribution Eq. 1 applies in any case conditional on the observed response marginal, no matter by which specific processing mechanism the hit and false alarm rates were generated in the first place. For example, in order to apply the conditional analysis based on Eq. 1 it would be irrelevant if the observed rates were generated by an underlying continuous strength model, for example, with normally or logistically distributed internal representations, or by a discrete-state model (Macmillan & Creelman, 2005, Ch. 4), or by yet another processing mechanism (e.g., Schwarz, 1992). As the log likelihoods obtained from independent tables add up, the conditional analysis is easily extended beyond single-point designs to series of  2 × 2 tables characterized by a common odds ratio, as would be obtained by collecting from one observer several independent points on an ROC of the form shown in Fig. 4 (e.g., Agresti, 2013, Ch. 6; Fleiss et al., 2003, Ch. 10; Gart, 1970; Pawitan, 2013, Ch. 10).

### Statistical power: Comparison of conditional and unconditional approaches

It has been observed in various statistical contexts that techniques based on conditional inference tend to be slightly more conservative, relative to unconditional approaches (Agresti, 2013, Ch. 3.5 and 16.6; Choi et al., 2015). To evaluate to which degree this observation also holds for evaluating performance in the signal detection design underlying Table 1, we compared the statistical power of the two approaches. To this end, we used the data generation model conforming to the “home ground” of traditional SDT—that is, the equal variance normal distribution model. Specifically, we used the double-binomial YN design underlying Table 1 for an unbiased observer (c = 0) and with *n*_*1*_ = *n*_*0*_ = 40 trials per stimulus. For these numbers, the assumptions underlying the derivation of the approximate standard error *SE* ($\hat{d^{\prime }}$) would usually be considered to be satisfied (e.g., Kadlec, 1999). We first explicitly computed for any of the 41 × 41 possible combinations of observed numbers of hits *x* and false alarms *m − x* if the value of *ψ* = 0 is contained in the 95% confidence interval described above, as obtained by inverting the two-sided test based on Eq. 1 (e.g., Agresti & Min, 2001; Agresti, 2013, Ch. 16). In a second step, these conditional outcomes, given *x* and *m*, were then weighted according to the double-binomial sampling model and summed to get the overall probability of rejecting the hypothesis of *d* ′  = 0.

**Fig. 5:**
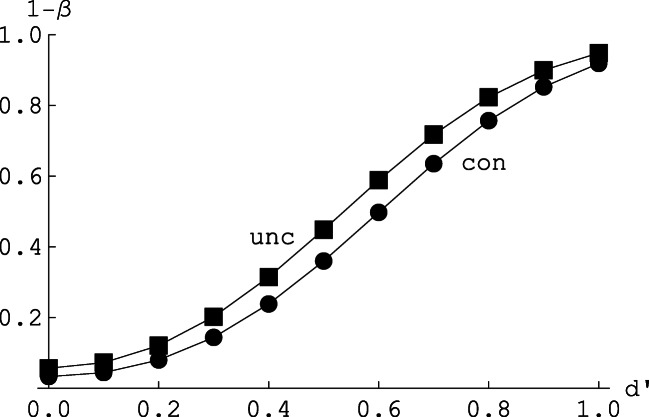
The power function for the conditional (con; lower curve and circles) and unconditional (unc; upper curve and squares) approach. Abscissa: true underlying sensitivity *d′* in the standard equal variance normal distribution model. Ordinate: probability 1 − β to reject the hypothesis of no sensitivity, *d′* = 0. Based on independent double-binomial sampling of *n*_*1*_ = 40 signal trials and *n*_*0*_ = 40 noise trials, and assuming no bias (c = 0)

Similarly, we determined for any of the 41 × 41 combinations of *x* and *m − x* in Table 1 if the value of *d′* = 0 was contained in the 95% confidence interval, as obtained by the standard unconditional approach, $\hat{d^{\prime }}$ ±1.96·*SE* (*d′*) (e.g., Macmillan & Creelman, 2005, Ch. 13). Note that the standard estimate of *d′* is undefined if either the observed hit rate *H* or the observed false alarm rate *F* is equal to zero or one. We followed the convention to replace rates of zero by 1/(2n) and rates of one by 1 − 1/(2n); relative to other conventions (see Hautus, 1995; Kadlec, 1999; Miller, 1996; Rotello et al., 2008) this choice had very little effect because for an unbiased observer with *n*_*1*_ = *n*_*0*_ = 40 trials the probability to observe rates of zero or one is extremely small. Again, these conditional outcomes, given *x* and *m*, were then weighted according to the double-binomial sampling model. Thus, the results shown in Fig. 5 are explicitly computed exact results, not simulations.

The following main results shown in Fig. 5 stand out. First, for any *d′* > 0, the unconditional approach is in fact more powerful. Second, the power advantage of the unconditional approach is rather small, about 0.07 in terms of the *d′* metric, describing the horizontal shift of the two power curves. Third, the conservativeness of the conditional approach means that the actual α−error (3.3%) is below the nominal α−level of 5%, whereas for the unconditional approach the actual α−error is 5.7%, that is, about 14% larger than the nominal level (for systematic power simulations of the normal distribution model, see Rotello et al., 2008). It is therefore an open question if the power differences shown in Fig. 5 merely reflect a downstream consequence of the different actual α−errors. To address this point, we increased the factor 1.96 for the standard confidence interval in the unconditional approach until the actual α−error was just equal to the value of α = 3.3% for the conditional approach. This required a factor of 2.20, and with the actual α−errors equated in this manner, the two power curves were indistinguishable. It is thus fair to conclude that the conditional approach is as powerful as the unconditional approach, at least when the actual α−errors involved are equated in the manner indicated.

### General discussion

The conditional and the unconditional approach represent two prominent alternative statistical frameworks to analyze data in the format of Table 1 (Agresti, 2013; Cox & Snell, 1989; Pawitan, 2013). For example, in order to compare two observed relative frequencies generations of psychologists (e.g., Hays, 1963, Ch. 17; McNemar, 1962, Ch. 13) have used Fisher’s exact test, arguably the most prominent conditional statistical test, corresponding to the null case of λ = 1 in Eq. 1. Similarly, classical probabilistic models in item response theory are explicitly based on a conditional inference framework (Rasch, 1966). Against this background, it is surprising that in evaluating detection performance the conditional approach has played no role so far. The present note describes the conceptual framework on which the conditional approach to evaluating detection performance rests, and illustrates some technical aspects of its application in the context of the YN design of SDT. In the following we aim at a balanced discussion of some pros and cons of this approach.

A central feature of the conditional approach is that it avoids the dependence on nuisance parameters, such as, for example, the traditional response criterion measure c, by conditioning on the actually observed response marginal. In many contexts, this strategy seems reasonable from a perceptual or cognitive point of view. For example, Kantner and Lindsay (2012) presented strong evidence that the response bias shown by an observer resembles a trait-like predisposition that is largely independent of the specific manipulations that separate signal from noise, and on which the interest of most researchers typically focuses. Reasoning on the basis of the actually observed response marginal, the conditional approach relies on an exact and explicit probabilistic basis, Eq. 1, for inference, thereby avoiding linearizing approximations, or appeal to asymptotic large-sample convergence in distribution. The basis of Eq. 1 means that all well-established analytical tools of standard likelihood theory (e.g., Morgan, 2009; Pawitan, 2013) apply, and that the approach remains valid even for very small numbers of trials, which is especially valuable in applied contexts where the number of trials per condition and observer is typically small. Note that Eq. 1 remains valid also for extreme observations such as *F* = 0 or *H* = 1; in these cases the likelihood function is strictly increasing so that no finite conditional maximum likelihood estimate of λ exists but confidence intervals will still give finite lower limits for λ.

It is informative to compare these aspects to the unconditional approach. For the special case of an unbiased observer (i.e., assuming that c = 0), Miller (1996) first showed, starting from a given value of the true underlying *d′* , how to derive numerically the exact sampling distribution of $\hat{d^{\prime }}$ from the basic double-binomial sampling model. In contrast, in applied studies, inference has to work backwards from the observed value of $\hat{d^{\prime }}$ to probabilistic conclusions about *d′* . To derive confidence intervals for *d′* , Miller (1996, Eq. 12) inverted his numerical results for the exact sampling distribution of $\hat{d^{\prime }}$ as computed under the equal variance normal distribution model. His approach rests on the a priori assumption of c = 0 regarding the nuisance parameter c; by comparison, the conditional approach achieves this elimination by conditioning on the actually observed response marginal. As shown in the Introduction, in the general case the unconditional confidence intervals depend on the value of c when (as would usually be the case) no a priori knowledge about *c* is available. It is clearly possible to generalize Miller’s (1996, Eq. 12) approach, for example, by deriving two-dimensional confidence regions for (*d, c*) defined by equal-likelihood contours; however, such an approach would no longer be exact but have to rely on asymptotic large-sample distribution theory involving the usual approximation that −2 times the log likelihood ratio is χ^2^−distributed (e.g., Pawitan, 2013, Ch. 4.3; Morgan, 2009, Ch. 4).

A further central feature of the conditional approach is that it does not rely on particular assumptions regarding the underlying perceptual or cognitive processing mechanisms, such as specific continuous strength or discrete state models (cf. Macmillan & Creelman, 2005, Ch. 4; Rotello et al., 2008; Schwarz, 1992). The only assumptions required are the independence of trials, and the across-trials constancy of the true underlying probabilities π_*H*_, π_*F*_. In this minimal framework, the conceptual hypothesis of no sensitivity essentially reduces to the simple hypergeometric urn model described in the Introduction. Note that, in contrast, the standard error *SE*($\hat{d^{\prime }}$) in the traditional unconditional analysis (Gourevitch & Galanter, 1967, Eq. 6; Macmillan & Creelman, 2005, Eq. 13.4) depends on the specific assumption of normally distributed internal representations.

These advantages have to be balanced against features of the conditional framework which, at least in some contexts, represent disadvantages relative to the unconditional approach. First, precisely because the conditional framework eliminates the dependence on nuisance (i.e., bias) parameters by conditioning on the observed response marginal, it cannot provide an explicit measure of response bias. Therefore, in contexts where it is important to derive explicit bias measures the unconditional approach is the obvious choice. Second, the unconditional approach has slightly more statistical power to detect a given level of sensitivity. For scenarios typical of applied research the difference in power is minuscule (see Fig. 5); it is bought at the price of an α−error that is larger than that for the more conservative conditional approach, and is absent for typical scenarios as shown in Fig. 5 if the actual α−levels involved are equated. Third, the minimalistic set of conceptual and technical assumptions required for the conditional framework may instead be seen as a limitation. Many applied studies using single-point YN designs aim simply at comparing rates of hits and false alarms, whereas others explicitly seek to test and compare specific information processing models differing in, for example, their assumptions about continuous vs. discrete stimulus representations (e.g., Macmillan & Creelman, 2005, Ch. 4). The conditional framework essentially compares probabilities and evaluates their relation in terms of their (log) odds ratio but it remains mute with respect to how these probabilities are generated in terms of more basic perceptual or cognitive processing mechanisms.

The present note focuses on analyses at the level of an individual observer. This is in line with the fact that in many contexts, SDT is applied to single, or to a few individual cases of specific interest, for example, in medical and clinical studies involving rare diseases or specific conditions (e.g., Kostopoulou et al., 2019; O’Connor et al., 2003), in research involving a few trained animals (Blough, 2001), in legal case studies (Scurich & John, 2011), in research involving subjects claiming to be exceptionally sensitive to, for example, electromagnetic fields (Köteles et al., 2013), in linguistic studies of grammaticality judgments (Huang & Ferreira, 2020), or in case studies of suspected malingering (Hiscock & Hiscock, 1989; Merten & Merckelbach, 2013). The conditional framework of Eq. 1 is as well easily applicable to studies involving a larger number of cases for each of whom an individual estimate of *ψ* is derived, or to studies involving a single observer doing relatively few trials. Beyond the level of individual subjects the conditional framework suggests a basic metric (i.e., the log odds ratio, *ψ*) that is statistically well-understood, and clearly lends itself to higher-level meta-analytic aggregation of primary cases, or to the comparison and aggregation of a series of tables such as Table 1 (for background, see Agresti, 2013, Ch. 6; Fleiss et al., 2003, Ch. 10; Gart, 1970; Pawitan, 2013, Ch. 10).

In conclusion, it seems fair to expect that the conditional approach to evaluating detection performance will prove useful in contexts in which closely related well-established tools based on conditional inference such as Fisher’s exact test have since long been valuable and prominent. These contexts include single-point detection studies in which the number of signal and noise trials are small, the assumption of specific strong processing models seems unwarranted, and the analysis of response bias is not of main interest. For these scenarios, the conditional framework may profitably not replace, but complement the more traditional unconditional approach to evaluating detection performance.

## Footnotes

1 The variant of the model based on logistic distributions is discussed in more detail below.

2 Estimates of *d′* and *c* are independent only if the observer is unbiased (i.e., when *c* = 0).

3 We set aside Bayesian approaches (Agresti, 2013, Ch. 3.6) at this point, which have so far not played a prominent role in applied studies evaluating detection performance; for an instructive example, see Hyett et al. (2014).

4 We neglect the fact that the standard estimate of *d′* is in fact slightly biased; see, for example, Hautus (1995), Kadlec (1999), Miller (1996), and Verde et al. (2006).
